# Looking for a Cultured Surrogate for Effectome Studies of the Clubroot Pathogen

**DOI:** 10.3389/fmicb.2021.650307

**Published:** 2021-05-28

**Authors:** Melaine González-García, Edel Pérez-López

**Affiliations:** ^1^Department of Plant Sciences, Faculté des Sciences de l'agriculture et de l'alimentation (FSAA), Université Laval, Québec, QC, Canada; ^2^Centre de recherche et d'innovation sur les végétaux (CRIV), Université Laval, Québec, QC, Canada; ^3^Institut de biologie intégrative et des systèmes (IBIS), Université Laval, Québec, QC, Canada; ^4^Centre de recherche en sciences du végétal (Centre SÈVE), Fonds de recherche du Québec - Nature et technologies (FRQNT), Québec, QC, Canada

**Keywords:** clubroot, *Plasmodiophora brassicae*, Brassicas, surrogate, unculturable plant pathogen, effector biology, MPMI

## Brassica-*Plasmodiophora Brassicae*: a Challenging Pathosystem

Clubroot is a devastating disease infecting roots of plants of the Brassicaceae family and responsible for 10–15% yield reduction on a global scale (Botero et al., 2019). The clubroot pathogen, *Plasmodiophora brassicae*, is an obligate intracellular biotroph that belongs to the class Phytomyxea within the eukaryote supergroup Rhizaria, one of the least studied groups of eukaryotes (Burki et al., 2010). The life cycle of *P. brassicae* starts in the soil, where resting spores, which can remain viable for up to 20 years, germinate in response to the presence of plant hosts and initiate primary infection. During secondary infection, susceptible plants develop galls that disrupt water and nutrient uptake, leading to wilting, stunting, and, in some instances, death of the infected plant (Dixon, 2006, 2009). The mechanisms by which the clubroot pathogen can escape plant immunity in the susceptible host and induce the root galls characteristics of the disease is still unknown (Pérez-López et al., 2018). Based on what we know about biotrophic pathogens from other evolutionary groups, effector proteins might play a key role in mediating *P. brassicae* pathogenicity (Jones and Dangl, 2006). Prediction of effectors based on conserved motifs has been very challenging for this pathogen, mainly because of unique features inherent to *P. brassicae*, including its inability to grow in axenic cultures (Chen et al., 2019; Pérez-López et al., 2020). The first *P. brassicae* effector characterized was Pro1, a serine protease that may play a key role in stimulating germination of *P. brassicae* resting spores (Feng et al., 2010). The methyltransferase *Pb*BSMT was the second *P. brassicae* effector characterized (Ludwig-Müller et al., 2015), an effector that allows the pathogen to mimic regulation of salicylic acid within the susceptible host (Bulman et al., 2018; Djavaheri et al., 2019). The third and last *P. brassicae* effector characterized was the cysteine protease inhibitor SSPbP53, which plays a key role in protecting effectors from host apoplastic enzymes during secondary infection (Pérez-López et al., 2020, 2021). While some *P. brassicae* ubiquitin ligases have been functionally characterized, their role in pathogenicity has not been elucidated (Yu et al., 2019). This scarcity of information about *P. brassicae* effectors stresses the need to find other ways to assess more effector candidates to better understand the etiology of the clubroot pathogen. Luckily for us, the researchers aiming a better understanding of the clubroot pathogen, thale cress (*Arabidopsis thaliana*), the model plant in molecular biology, is susceptible to most of the *P. brassicae* pathotypes and its use can facilitate our search for new methodologies and the interpretation of the results.

## Cultured Surrogates to Characterize Unculturable Plant Pathogens

Several strategies have been followed to study the effector repertoire of unculturable plant pathogens. Two of them are based on the heterologous expression of the effector candidates: (*i*) in the host plant or the model plant *Arabidopsis thaliana*, and (*ii*) by a related or unrelated cultured microorganism to evaluate the role of the effector in pathogenicity. The first strategy has allowed the characterization of several phytoplasma effectors such as SAP54, an effector that generates a short circuit between two key pathways of the host to alter plant development and to promote insect feeding of the infected plant to increase the propagation of the pathogen (MacLean et al., 2014; Tomkins et al., 2018). This strategy, although successful, is long and laborious and, for those effectors that do not produce a visible phenotype in the transgenic plant, as with SAP54, further analyses like RNAseq should be performed (dos Santos et al., 2020).

The use of a surrogate culturable microorganism represents an interesting approach to facilitate the understanding of the clubroot pathogen. It has been exploited (1) to characterize the flavescence doree phytoplasma variable membrane protein VmpA, using *Spiroplasma citri* (Renaudin et al., 2015) as surrogate, (2) to characterize a “*Candidatus* Liberibacter asiaticus” cysteine protease inhibitor, using *Pseudomonas syringae* pv. *tomato* (Clark et al., 2018), and (3) to characterize a “*Candidatus* Liberibacter asiaticus” glyoxalase using the culturable *Liberibacter crescent* (Jain et al., 2017), among other examples. In the previous studies, a culturable bacterium was used as a surrogate of an unculturable one, but evolutionary distant microorganisms have also been used. For instance, *Pseudomonas syringae* pv. *tomato* (*Pst*DC3000) served as surrogate for the unculturable oomycete *Hyaloperonospora arabidopsis* (Hpa) (Fabro et al., 2011; Goritschnig et al., 2012), a breakthrough that allowed, for the first time, the identification and characterization of *Hpa* effectors suppressing plant immunity (Fabro et al., 2011) and eliciting effector-triggered immunity (ETI) (Goritschnig et al., 2012).

## The Best Surrogate for *P. Brassicae*

The use of a culturable surrogate to characterize *P. brassicae* proteins has been previously attempted. Recently, *Magnaporthe oryzae* was the used to identify the role of the cyclophilin *Pb*CYP3 on pathogenicity (Singh et al., 2018). Although this is a sensible approach, a negative point of using *M. oryzae* as a *P. brassicae* surrogate is that it is a rice pathogen with a high degree of host specificity (Couch et al., 2005). This makes it unreliable to characterize *P. brassicae* proteins with a suspected target in plants.

*Pseudomonas* spp. has been exploited as a model organism allowing conceptual advances in understanding many key aspects of plant-microbe interactions. Recent technical advances such as the development of an image-based system to quantify plant immunity (Laflamme et al., 2016) and a high-throughput seedling screening of plant immunity (Martel et al., 2020), both using the *Arabidopsis*-*Pseudomonas* pathosystem, could facilitate the use of *P. syringae* as a surrogate to understand *P. brassicae*. Like *P. brassicae, P. syringae* is a biotrophic plant pathogen and can infect Arabidopsis, two more elements reinforcing *Pseudomonas* spp. as a good surrogate for the clubroot pathogen. Another milestone recently achieved in the study of *P. syringae* as a model organism was the identification of the *P. syringae* pan-genome effectors triggering ETI in Arabidopsis, expanding the number and protein families previously known to elicit ETI (Laflamme et al., 2020). Among those protein families able to elicit ETI during *Arabidopsis*-*Pseudomonas* interaction, proteases, lipases, carbohydrate esterases, ADP-ribosyltransferases, and GTPase-activating proteins were identified (Laflamme et al., 2020). Members of all those protein families are found in the *P. brassicae* genome, without transmembrane domains and functional signal peptide (Pérez-López et al., 2020), supporting the untested theory that resistance induced by ETI elicited by *P. brassicae* effectors and mediated by NLR receptors, is the base of the resistance observed for some canola cultivars (Hejna et al., 2019). Using *Pseudomonas* spp. as a surrogate would allow to deliver *P. brassicae* effectors individually and use them as probes to identify resistance proteins mediating ETI.

The use of a prokaryote as surrogate for an eukaryotic pathogen has however several disadvantages. These include incorrect protein folding, incorrect post-translational modifications, and the simple fact that some strategies used by eukaryotic plant pathogens are not used by prokaryotes (van den Burg et al., 2004; Macek et al., 2019). How can this problem be solved? A simple solution would be to explore another culturable surrogate. In a recent study, our group identified two effectors, *Pb*ChiB2 and *Pb*ChiB4, able to bind to chitin oligomers *in vitro*, and suppress chitin-triggered immunity (Muirhead and Pérez-López, 2020). These effectors were able to bind chitin through a CBM18 domain as previously described for the soil-borne pathogen *Verticillium dahliae* (Volk et al., 2020). Like *P. brassicae, V. dahliae* can affect Arabidopsis, with an hemibiotrophic lifestyle. Besides, *V. dahliae* can be transformed to express clubroot pathogen effectors (Gao et al., 2019). A common advantage with the use of *P. syringae* and *V. dahliae* is that the typical symptomatology induced by both plant pathogens is detected in the leaves, allowing the use of visual assessment to study the effect of *P. brassicae* candidate effectors on pathogenicity.

## Conclusions

**Figure 1:**
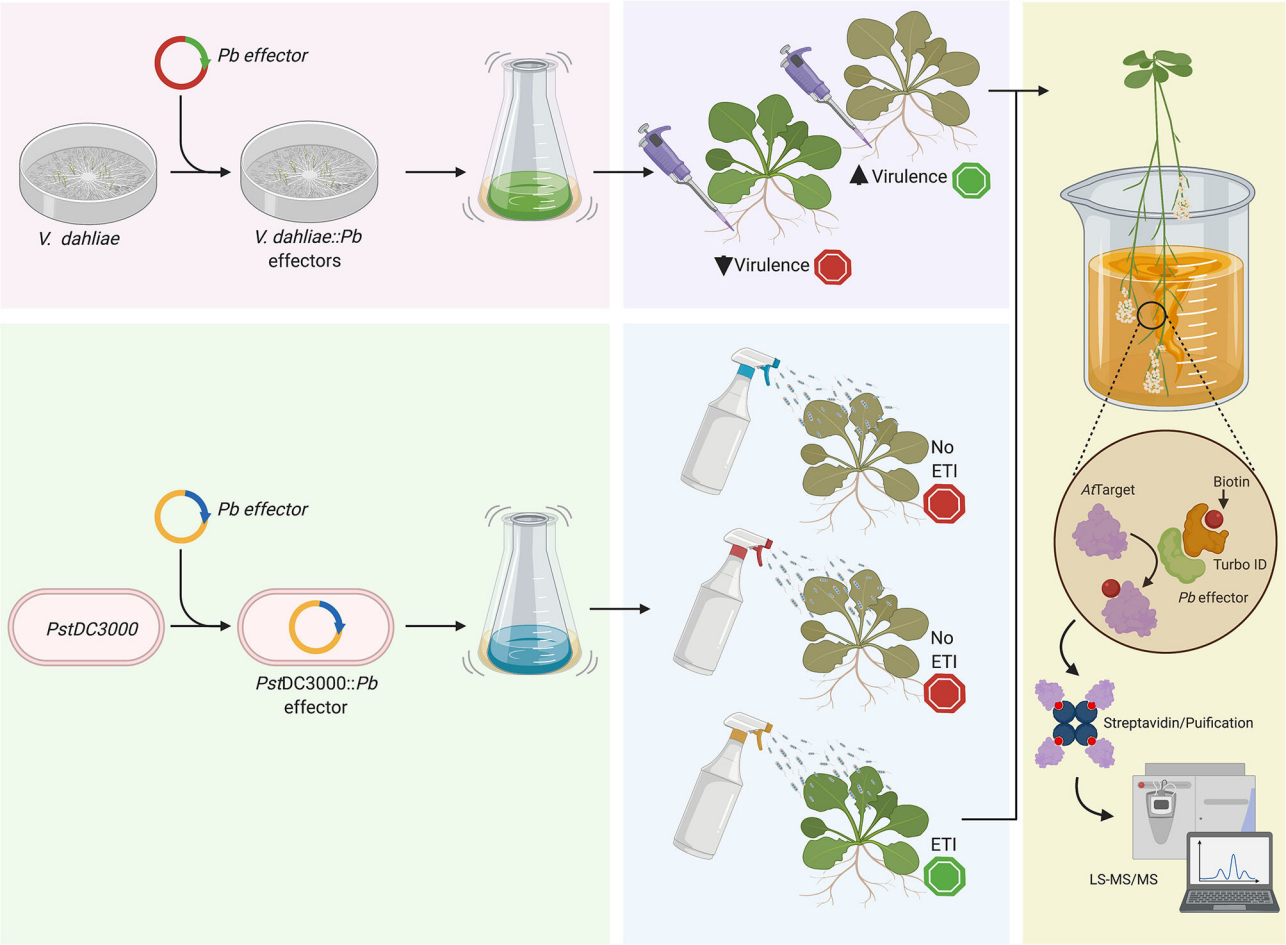
Schematic representation of the use of a cultured surrogate plant pathogen to study and characterized *P. brassicae* candidate effectors. Here we proposed the use of (*i*) *V. dahliae* to, for example, complement deleterious mutations like the case discussed in the main text about the chitin-binding effectors, and (*ii*) *P. syringae* pv. *tomato* (*Pst*DC3000), which can be used to screen effectors eliciting effector-triggered immunity. After the initial step selecting candidate effectors with a role on pathogenicity through the assessment of foliar symptomatology, we could identify the target in the plant host using a protein-protein approach like BioID as previously described Khan et al. (2018). Created with BioRender.com.

The complexity and uniqueness of the Brassicas-*P. brassicae* pathosystem do not allow us to select only one cultured surrogate for effectome studies. Is that complexity the reason why there is so little known about the clubroot pathogen biology and the mechanisms used to infect the plant and to suppress and/or escape plant immunity. For this reason, we believe that different approaches, including the use of versatile surrogates such as *P. syringae* and *V. dahliae*, can provide a multi-prong solution to further the characterization of *P. brassicae* effectors and their targets in plants (Figure 1), although questions like how the effectors are secreted to the plant cell are still to be answered. Through the application of such novel approaches, we can hope to make advancements toward the development of resistant germplasm that can mediate losses to this important pathogen.

## Author Contributions

All authors listed have made a substantial, direct and intellectual contribution to the work, and approved it for publication.

## Conflict of Interest

The authors declare that the research was conducted in the absence of any commercial or financial relationships that could be construed as a potential conflict of interest.
